# Supplementary material for: MoS2/SnS/CoS Heterostructures on Graphene: Lattice-Confinement Synthesis and Boosted Sodium Storage
Source: Molecules. 2023 Aug 9;28(16):5972. doi: 10.3390/molecules28165972 (PMC10458794; doi:10.3390/molecules28165972)
Supplement: Supplementary file 1 [file molecules-28-05972-s001.zip › molecules-2519609-supplementary.pdf]

SUPPLEMENTARY MATERIALS

MoS_2/SnS/CoS Heterostructures on Graphene: Lattice-Confinement Synthesis and Boosted Sodium Storage

Ruyao Zhang [1,†], Yan Dong [1,†], Yu Su [1], Wenkai Zhai [1] and Sailong Xu [2,*]

[1] State Key Laboratory of Chemical Resource Engineering,
Beijing University of Chemical Technology, Beijing 100029, China;
2022210730@buct.edu.cn (R.Z.); dongyan2232021@163.com (Y.D.);
suyu_yh@163.com (Y.S.); 2021210568@buct.edu.cn (W.Z.)

[2] Quzhou Institute for Innovation in Resource Chemical Engineering,
Quzhou 324000, China

* Correspondence: xusl@buct.edu.cn

† These authors contributed equally to this work.

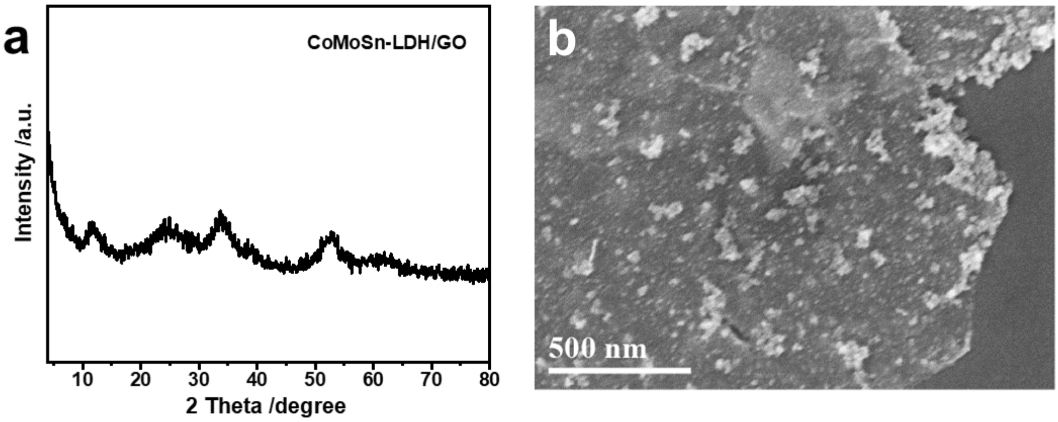

Figure S1. The CoMoSn-LDH/GO precursor: (a) XRD pattern, (b) SEM image.

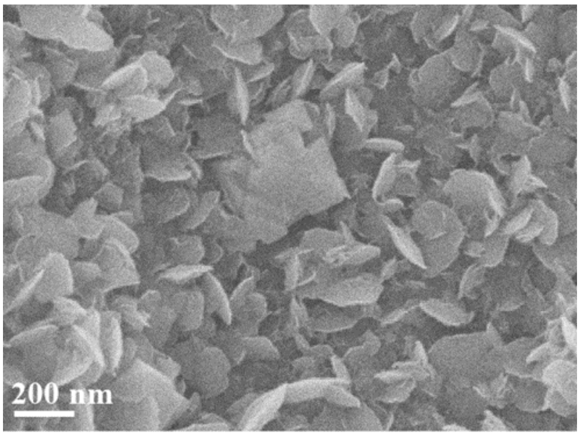

Figure S2. The SEM image of the MoS_2/SnS/CoS@rGO composite.

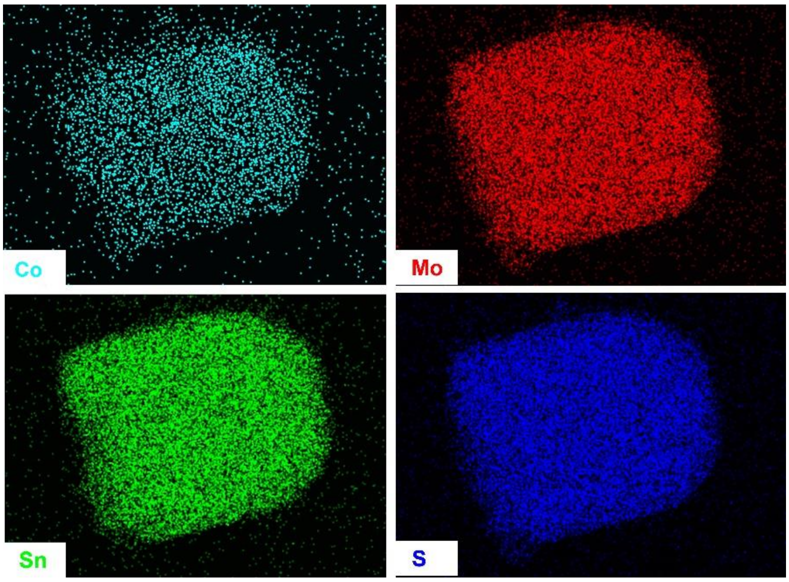

Figure S3. The EDS Mapping images of the Co, Mo, Sn and S elements in the MoS_2/SnS/CoS@rGO composite.

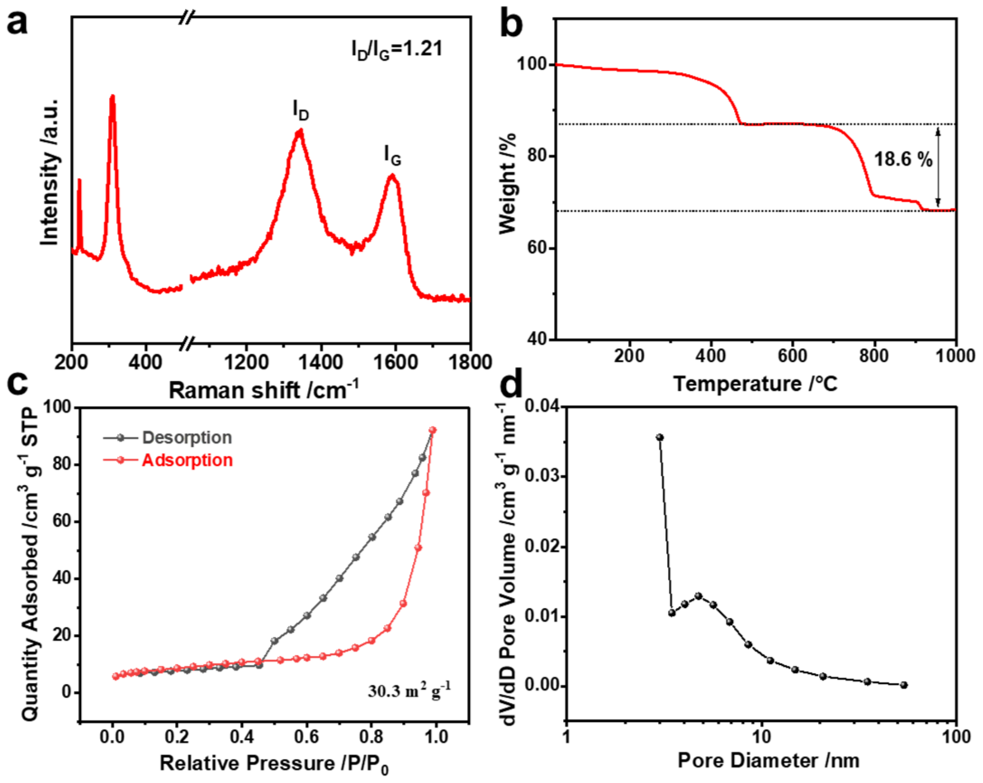

Figure S4. The MoS_2/SnS/CoS@rGO composite. (a) Raman spectroscopy, (b) TG curve, (c) N_2 adsorption and desorption curves and (d) the corresponding pore size distribution.

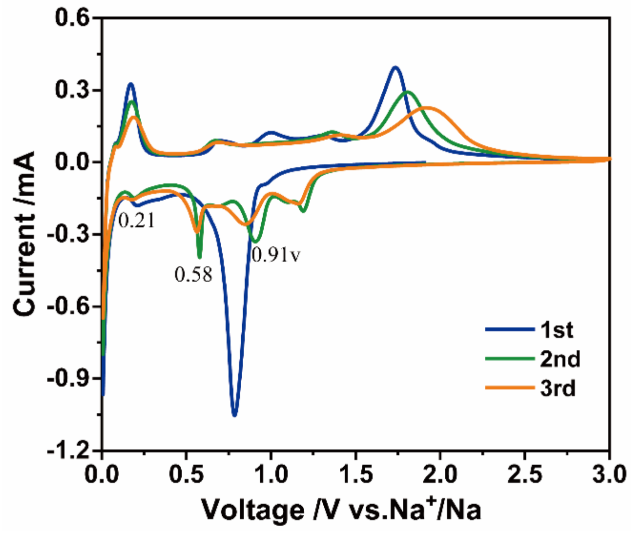

Figure S5. The initial three CV curves of MoS_2/SnS/CoS@rGO at 0.1 mV s^{-1}.

Table S1. Comparison of cycling performances between our MoS_2/SnS/CoS@rGO and the CoS-based anode nanomaterials for SIBs.

**Anode nanomaterials**	**Current density ($A\ g^{-1}$)**	**Capacity ($mA\ h\ g^{-1}$)**	**Cycles**	**Reference**
MoS_2/CoS@CC	0.5	605	100	*Nanoscale*, 2023, 15, 6822
CoS/Co_9S_8/N,S-C	0.1	409.2	100	*ACS Nano*, 2017, 11, 12658
CoS@SnS/C sphere	2	368	1000	*ACS Appl. Energy Mater.* 2021, 4, 5574
FeS_2/CoS	1.0	900	610	*J Solid State Electrochem,* 2023, DOI: 10.1007/s10008-023-05544-4
CoS/Cu_2S@C-NC	2.0	435.3	1000	*Small* 2023, 19, 2302706
CuS@CoS_2	5.0	304	500	*Angew. Chem. Int. Ed.* 2019, 58, 7739
NiS_2@CoS_2	0.1	848	—	*J Colloid Interf. Sci.* 2023, 633, 480
CoS-HNP@CFs	0.1	670	—	*ACS Appl. Mater. Interfaces* 2018, 10, 40531
CoS /NC@VS_4	1.0	307	700	*J Colloid Interf. Sci.* 2022, 625, 41
MoS /CoS-rGO	5.0	215	50	*J Colloid Interf. Sci.* 2020, 575, 42
CoS/SnS/C	0.1	463	100	*J Colloid Interf. Sci.* 2022, 609, 403
MoS_2/SnS/CoS@rGO	0.1	620	100	**This work**
	5.0	304	1000	

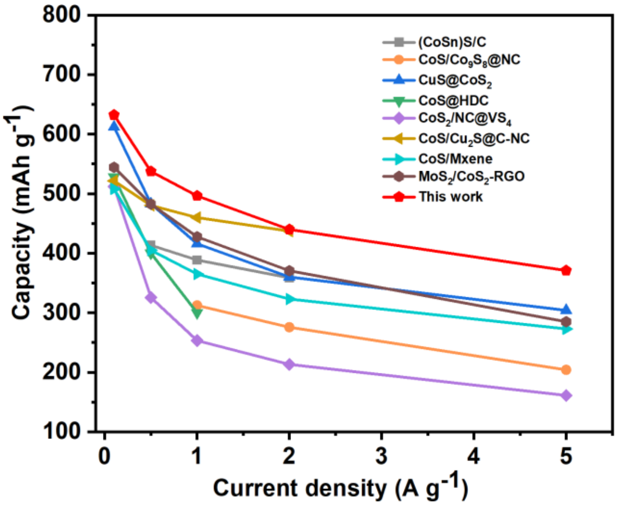

Figure S6. Comparison of rate capability between MoS_2/SnS/CoS@rGO and those reported sulfides that are listed in Refs. [38–40,53–57] in the text.

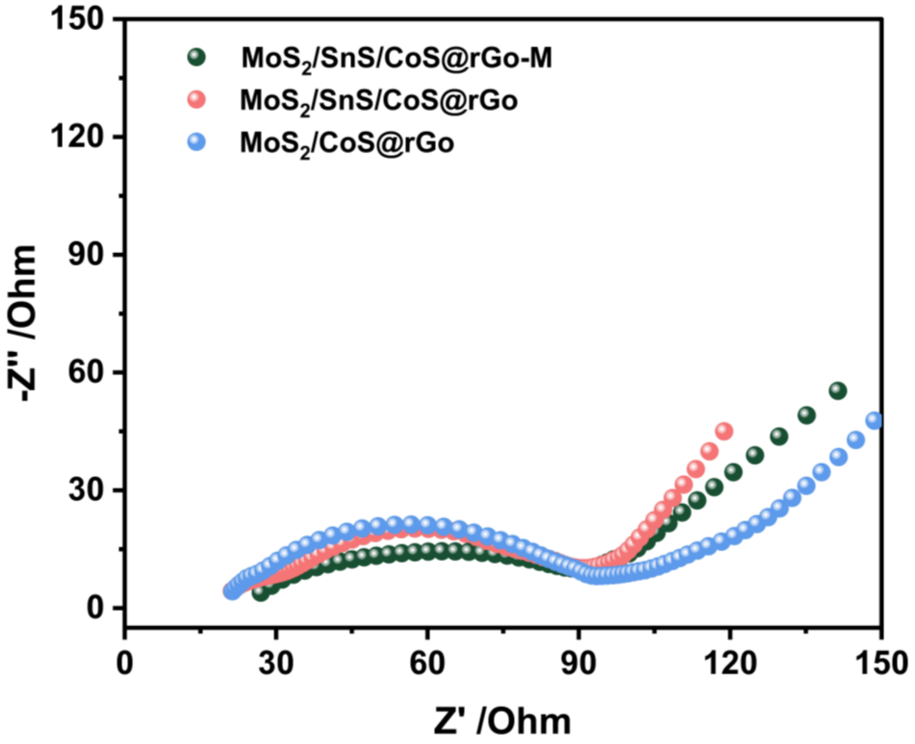

Figure S7. Nyquist plots of MoS_2/SnS/CoS@rGO, MoS_2/CoS@rGO, and MoS_2/SnS/CoS@rGO-M.

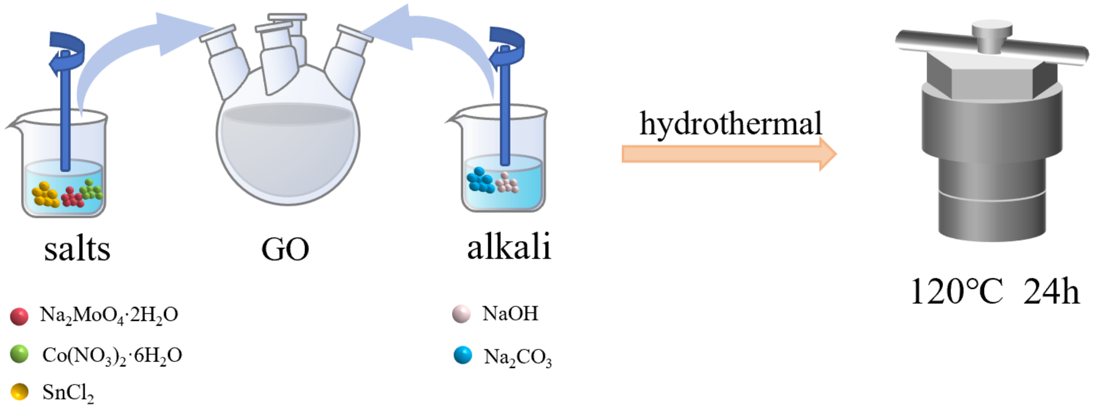

Scheme S1. Schematic illustration of the preparation of the CoMoSn-LDH/GO precursor.

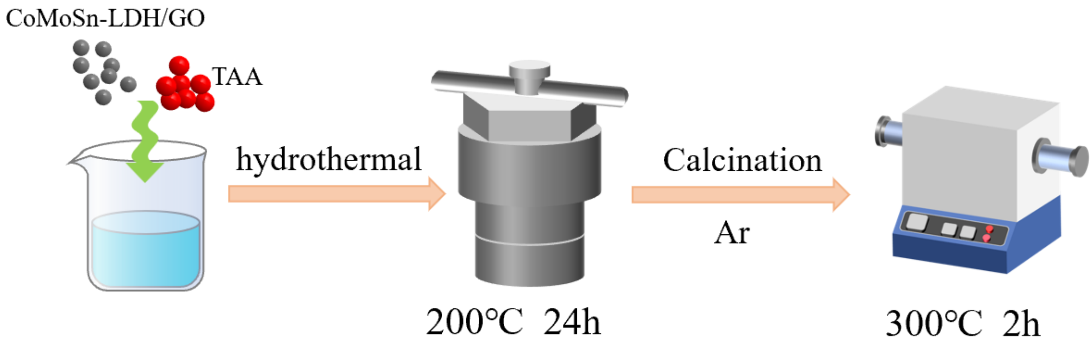

Scheme S2. Schematic illustration of the preparation of the MoS_2/SnS/CoS@rGO composite.
